# The prevalence and correlates of pre-diabetes in middle- to older-aged Irish adults using three diagnostic methods

**DOI:** 10.1371/journal.pone.0253537

**Published:** 2021-06-25

**Authors:** Kate Junker, Claire M. Buckley, Seán R. Millar, Sinéad Flynn, Janas M. Harrington, Patricia M. Kearney, Ivan J. Perry

**Affiliations:** HRB Centre for Health and Diet Research, School of Public Health, University College Cork, Cork, Ireland; Government College University Faisalabad, PAKISTAN

## Abstract

**Background and objectives:**

Type 2 diabetes is a leading cause of death and disability worldwide and pre-diabetes is a strong predictor of diabetes development. To date, studies estimating the prevalence of pre-diabetes in the Irish population are sparse and conflicting. Monitoring the prevalence of pre-diabetes and a knowledge of associated factors is required to inform policies and to prevent development of type 2 diabetes. Therefore, this research examined the prevalence and correlates of pre-diabetes in a sample of middle- to older-aged Irish adults using three different methods for diagnosis.

**Materials and methods:**

The Mitchelstown Cohort Rescreen (2016/17) was a follow-up, cross-sectional study of the Mitchelstown Cohort Study (2010/11). 1,378 participants were recruited from a random sample of patients attending a single primary care centre. Pre-diabetes was defined using three diagnostic criteria: American Diabetes Association (ADA) glycated haemoglobin A_1c_ (HbA_1c_) cut-offs between 5.7%–6.4% (39–46 mmol/mol), World Health Organization International Expert Committee (WHO-IEC) HbA_1c_ cut-offs between 6.0%–6.4% (42–46 mmol/mol) and ADA fasting plasma glucose (FPG) cut-offs between 5.6–6.9 mmol/l. Univariate and multivariable logistic regression analyses were used to determine factors associated with pre-diabetes.

**Results:**

The prevalence of pre-diabetes was found to be 43.9% (95% CI: 41.2%─46.5%), 14.5% (95% CI: 12.7%─16.5%) and 15.8% (95% CI: 13.9%─17.8%) according to HbA_1c_ ADA, HbA_1c_ WHO-IEC and FPG ADA definitions, respectively. Depending on diagnostic method, factors associated with pre-diabetes in univariate analyses included sex, age, marital status, health rating, education and poor diet quality. In multivariable analysis, subjects classified by the FPG ADA pre-diabetes criterion displayed the least optimal metabolic profile defined by overweight and obesity (OR = 2.88, 95% CI: 1.53–5.43), hypertension (OR = 2.27, 95% CI: 1.51–3.40) and low high-density lipoprotein cholesterol concentrations (OR = 1.75, 95% CI: 1.07–2.87).

**Conclusions:**

The discordance between prevalence estimates according to method of diagnosis is concerning. A National Diabetes Prevention Programme is currently being developed in Ireland. Monitoring the prevalence of pre-diabetes over time will be important to assess the effectiveness of this programme. This study will inform national decision-makers on which definition of pre-diabetes to use for monitoring purposes.

## Introduction

Type 2 diabetes, a disease characterised by chronic hyperglycaemia, is a leading cause of death and disability worldwide [1–3]. The prevalence and burden of diabetes has increased considerably in world populations over recent years, driven primarily by rising levels of obesity and an ageing population [1, 3–8]. Type 2 diabetes is associated with an array of adverse health and well-being effects including diabetic retinopathy, diabetic nephropathy, diabetic neuropathy, cardiovascular disease [3], physical limitations, reduced quality of life and increased years lived with disability and years lost due to premature mortality [1, 9]. These consequences, as well as the considerable economic burden of spending related to diabetes [3, 10], demonstrate the public health importance of intervening in at-risk populations [9, 11].

Pre-diabetes, a condition defined by glycaemic profiles that are higher than normal but which do not meet thresholds for diabetes, is a strong risk factor for type 2 diabetes development [11–15]. Pre-diabetes is generally asymptomatic and may be defined by glycated haemoglobin A_1c_ (HbA_1c_) or fasting plasma glucose (FPG) [1, 2, 6, 7, 11, 15–17]. The American Diabetes Association (ADA) classifies pre-diabetes as HbA_1c_ levels between 5.7%─6.4% (39–46 mmol/mol) or FPG levels between 5.6─6.9 mmol/l [11, 16]. In 2011, the World Health Organization International Expert Committee (WHO-IEC) [18] recommended HbA_1c_ cut-offs between 6.0%–6.4% (42–46 mmol/mol) to define a pre-diabetic state [19].

However, according to research, those identified as having pre-diabetes using one method may not be the same as those detected using another [7, 9, 14, 20–24] and a number of studies have noted discordance between prevalence estimates using different diagnostic criteria [6, 9, 14, 21, 23]. In addition, while evidence suggests that HbA_1c_ may be a good marker of type 2 diabetes, there is considerable controversy as to whether it may also correctly identify individuals at increased future risk of diabetes [25]. Consequently, there is a lack of definitive evidence regarding which diagnostic method is superior and the choice of test and thresholds used are often at the discretion of individual clinicians [7, 26].

A 2016 literature review and meta-analysis found that among adults aged 18 years and over, the national prevalence of doctor diagnosed diabetes in the Republic of Ireland increased significantly from 2.2% in 1998 to 5.2% in 2015 (P for trend < .001), with the largest increase in prevalence being observed among older age groups [8]. In Ireland, screening high-risk patients for type 2 diabetes has been encouraged since the introduction of national guidelines for diabetes care in 2002 [8]. Importantly, accurate identification of subjects at risk of developing diabetes is important for prevention programmes, as having up-to-date estimates of the prevalence of pre-diabetes and related factors may help inform public health initiatives to prevent or delay the onset of type 2 diabetes [27].

Nevertheless, recent estimates of the prevalence of pre-diabetes in Ireland are conflicting. In 2015, a prevalence of 5.5% was reported using HbA_1c_ 5.7%─6.4% cut-offs in the Irish Longitudinal Study on Ageing (TILDA) [1]. This compares to a prevalence of 19.8% reported using data from the 2007 SLÁN Survey of Lifestyle, Attitudes and Nutrition which also used the HbA_1c_ ADA criterion [12]. The baseline analysis of our cohort demonstrated considerable discordance between estimates using two diagnostic criteria; among a middle-aged population in 2010/11, the prevalence of pre-diabetes using HbA_1c_ ADA cut-offs was found to 49.1% compared to a prevalence 11.5% using FPG [6]. Given the discrepancy between these figures, updated estimates are required.

Therefore, the aim of this study was to provide an updated estimate of the prevalence of pre-diabetes among middle- to older-aged adults in Ireland using three different methods for diagnosis and to determine factors related to pre-diabetes in a random sample of 1,378 Irish men and women aged 52–77 years of age.

## Materials and methods

### Study population

The Cork and Kerry Diabetes and Heart Disease Study (Phase II–Mitchelstown Cohort Study) was a single-centre, cross-sectional study conducted between 2010 and 2011. The primary aim of the study was to estimate the prevalence of major cardiovascular risk factors in a middle-aged population in Ireland and to determine the proportion of the population at high-risk. A population-representative sample was recruited from a large primary care centre in Mitchelstown, County Cork, Ireland. The Livinghealth Clinic serves a population of approximately 20,000, predominantly white subjects, with a mix of urban and rural residents. Stratified random sampling was employed to recruit equal numbers of men and women from all registered attending patients in the 46–73-year age group. In total, 3,807 potential participants were selected from the practice list. Following the exclusion of duplicates, deaths and subjects incapable of consenting or attending appointment, 3,051 were invited to participate in the study and of these, 2,047 (49% male) completed the questionnaire and physical examination components of the baseline assessment (response rate: 67%). Details regarding the study design, sampling procedures and methods of data collection have been reported previously [28].

A follow-up study was conducted between 2016 and 2017. The aim of the follow-up study was to provide an updated profile of cardiovascular health and related factors in an Irish middle- to older-aged population and to compare findings with those obtained during the 2010/11 baseline assessment. Surviving baseline participants (n = 1,981) were invited to attend for a re-screen; participants deemed unfit or too ill to take part by their GP were excluded. Protocols were similar to the baseline study and data were recorded for 1,378 patients.

Ethics committee approval conforming to the Declaration of Helsinki was obtained from the Clinical Research Ethics Committee of University College Cork (Mitchelstown Cohort, clinical trials.gov identifier NCT03191227). A letter signed by the contact GP in the clinic was sent out to all selected participants with a reply slip indicating acceptance or refusal. All subjects gave signed informed consent, including permission to use their data for research purposes. The Mitchelstown Cohort Study is GDPR compliant.

### General health, clinical and anthropometric data

Independent variables likely associated with pre-diabetes and type 2 diabetes were identified through the literature [1, 3, 4, 6]. A general health and lifestyle questionnaire assessed demographic variables, lifestyle behaviours and morbidity. Information on sex, age, marital status, health rating, education, medication use, presence of type 2 diabetes and tobacco/alcohol use was provided by participants. In this study, ‘current smoker’ was compared to ‘never/former smoker’. Alcohol consumption was measured in units of alcohol consumed on a weekly basis and was categorised into the following levels: (i) non-drinker, i.e. <1 drink per week; (ii) moderate drinker, i.e. between 1 and 14 drinks per week; and (iii) heavy drinker, i.e. >14 drinks per week [29]. For our analyses, these were then re-categorised as ‘high alcohol intake vs. ‘moderate/no alcohol intake’. Physical activity levels were measured using the validated International Physical Activity Questionnaire (IPAQ) [30] and were classified as ‘low level physical activity’ vs. ‘moderate or high’ levels.

Clinical measurements were taken by researchers who were thoroughly trained according to the study research protocols. Blood pressure (BP) was measured using an Omron M7 Digital BP monitor (Omron Healthcare Co. Ltd., Japan) on the right arm after a five-minute rest in a seated position. The average of the second and third measurements was used in analyses. Hypertension was defined as a systolic BP ≥140 mmHg or a diastolic BP ≥90 mmHg or use of prescription anti-hypertensive medications. Height was measured with a portable Seca Leicester height/length stadiometer (Seca, Birmingham, UK) and weight was measured using a portable electronic Tanita WB-100MA weighing scale (Tanita Corp, IL, USA). The weighing scale was placed on a firm flat surface and was calibrated weekly. Body mass index (BMI) was calculated as weight in kilograms divided by the square of height in meters. Study participants with a BMI ≥25 kg/m^2^ were considered to be overweight or obese [31].

### Dietary assessment

Diet was evaluated using a modified version of the self-completed European Prospective Investigation into Cancer and Nutrition (EPIC) Food Frequency Questionnaire (FFQ) [32], which has been validated extensively in several populations [33]. Adapted to reflect the Irish diet, the 150-item semi-quantitative FFQ used in the current study was originally validated for use in the Irish population using food diaries and a protein biomarker in a volunteer sample [34] and incorporated into the SLÁN Irish National Surveys of Lifestyle, Attitudes and Nutrition 1998, 2002 and 2007 [35–37]. The FFQ was also validated using a 7-day weighed food record completed in another Irish study (Lifeways Cross-generational Study), with reasonable agreement for fat, carbohydrate, and their components, and with lower agreement for protein [38].

The average medium serving of each food item consumed by participants over the last 12 months was converted into quantities using standard portion sizes. Food item quantity was expressed as (gm/d) and beverages as (ml/d). Based on the FFQ, the Dietary Approaches to Stop Hypertension (DASH) diet score was constructed. DASH diet scores ranged from 5–40. Lower scores represent poorer and higher scores represent better quality diet [39]. We classified poor diet quality as a DASH diet score in the bottom 40% for the study sample according to a method used in previous research [40].

### Biological analyses

Participants attended the clinic in the morning after an overnight fast (minimum 8 hours) and blood samples were taken on arrival. Triglyceride and high-density lipoprotein cholesterol (HDL-C) levels were measured on Olympus 5400 biochemistry analysers with Olympus reagents using standardised procedures and fresh samples (Olympus Diagnostica GmbH, Hamburg, Germany). Abnormal metabolic factors were defined as high triglycerides ≥1.7mmol/l and low HDL-C (<1.03 mmol/l in males or <1.29 mmol/l in females) [41].

### Pre-diabetes classification

Fasting glucose and HbA_1c_ levels were measured by the Cork University Hospital Biochemistry Laboratory. Glucose concentrations were determined using a glucose hexokinase assay (Olympus Life and Material Science Europa Ltd., Lismeehan, Co. Clare, Ireland) and HbA_1c_ levels were measured in the haematology laboratory on an automated high-pressure liquid chromatography instrument Tosoh G7 [Tosoh HLC-723 (G7), Tosoh Europe N.V, Tessenderlo, Belgium]. Pre-diabetes was defined using HbA_1c_ ADA cut-offs between 5.7%─6.4% (39–46 mmol/mol), HbA_1c_ WHO-IEC cut-offs between 6.0%–6.4% (42–46 mmol/mol) [19] and FPG ADA cut-offs between 5.6─6.9 mmol/l [16]. In this study, participants were classified as having type 2 diabetes if they had a HbA_1c_ level ≥6.5% (≥48 mmol/mol) or a FPG level ≥7.0 mmol/l [16]. Results for HbA_1c_ and FPG were available for 1,361 (98.8%) and 1,364 (99.0%) subjects, respectively. Participants with a self-reported diagnosis of type 2 diabetes, but who did not have positive test results for diabetes by either HbA_1c_ or FPG, were excluded from analyses (n = 25).

### Statistical analysis

Descriptive characteristics were examined according to normoglycaemia, pre-diabetes and type 2 diabetes. Categorical features are shown as percentages and continuous variables are displayed as a mean (plus or minus one standard deviation) or a median and interquartile range for skewed data.

Relationships between factors and pre-diabetes and type 2 diabetes (as defined using the HbA_1c_ or FPG thresholds mentioned above) were examined using univariate multinomial logistic regression with normoglycaemia defined by HbA_1c_ ADA, HbA_1c_ WHO-IEC or FPG ADA cut-offs being used as the reference category. Odds ratios (OR) and 95% confidence intervals (CI) are reported. Binary logistic regression analyses were used to determine multivariable relationships with pre-diabetes according to each diagnostic method. Candidate variables that had a P value of less than .2 in univariate analyses were entered into multivariable models. Forward elimination regression was used to build the models, with model fit determined using the likelihood ratio chi-square. All variables were adjusted for each other.

Statistical analyses were carried out using IBM SPSS Statistics 25 (IBM Corp., Armonk, NY, USA). Confidence intervals for prevalence rates were calculated using the VassarStats statistical website [42]. For all analyses, a P value (two-tailed) of less than .05 was considered to indicate statistical significance.

## Results

### Descriptive characteristics

**Fig 1:**
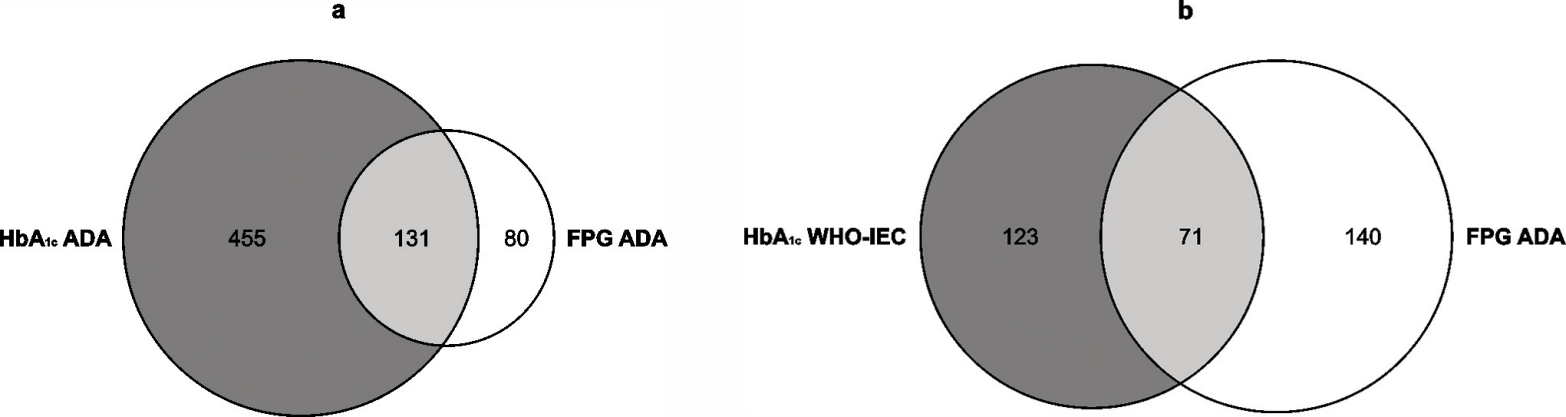
a and b. Levels of agreement between pre-diabetes classifications defined by HbA_1c_ or FPG. The figures show the levels of agreement between pre-diabetes classifications defined by HbA_1c_ or FPG for (a) HbA_1c_ ADA compared to FPG ADA and (b) HbA_1c_ WHO-IEC compared to FPG ADA.

Table 1 shows characteristics of the study population according to normoglycaemia, pre-diabetes and type 2 diabetes classifications. The prevalence of pre-diabetes in this sample was found to be 43.9% (95% CI: 41.2%─46.5%), 14.5% (95% CI: 12.7%─16.5%) and 15.8% (95% CI: 13.9%─17.8%) according to HbA_1c_ ADA, HbA_1c_ WHO-IEC and FPG ADA definitions, respectively. The levels of agreement between pre-diabetes classifications are shown in Fig 1A and 1B. Agreement between the HbA_1c_ ADA and FPG ADA (Kappa: 0.06; SE: 0.01) and the HbA_1c_ WHO-IEC and FPG ADA (Kappa: 0.11; SE: 0.02) pre-diabetes definitions was found to be poor.

**Table 1:** Characteristics of the study population according to glycaemic status by glycated haemoglobin A_1c_ (HbA_1c_) or Fasting Plasma Glucose (FPG).

Variable	Normoglycaemia			Pre-diabetes			Type 2 diabetes	
	HbA_1c_ ADA	HbA_1c_ WHO-IEC	FPG ADA	HbA_1c_ ADA	HbA_1c_ WHO-IEC	FPG ADA	HbA_1c_	FPG
	n = 651	n = 1043	n = 1038	n = 586	n = 194	n = 211	n = 99	n = 90
Male (%)	336 (51.6)	509 (48.8)	480 (46.2)	269 (45.9)	96 (49.5)	133 (63.0)	67 (67.7)	61 (67.8)
Age, years (median)	64.2 (59.8─68.4)	64.5 (60.2─68.8)	64.5 (60.2─68.9)	65.7 (61.2─70.1)	67.0 (62.3─71.2)	66.3 (61.5─70.7)	66.4 (63.4─69.7)	66.9 (64.1─70.0)
Age, ≥65 years (%)	294 (47.8)	488 (49.7)	486 (50.1)	306 (55.8)	112 (61.5)	122 (60.4)	62 (70.5)	58 (72.5)
Married or cohabiting (%)	517 (79.9)	826 (79.6)	824 (79.7)	447 (76.4)	138 (71.1)	151 (71.9)	78 (78.8)	69 (76.7)
Health rating, very good or good (%)	581 (89.7)	910 (87.5)	910 (87.9)	492 (84.0)	163 (84.0)	168 (79.6)	69 (69.7)	63 (70.0)
Primary education only (%)	130 (21.6)	222 (22.9)	208 (21.7)	140 (25.7)	48 (27.0)	62 (30.8)	32 (36.0)	31 (38.8)
Current smoker (%)	43 (6.6)	89 (8.6)	89 (8.6)	62 (10.6)	16 (8.2)	19 (9.0)	10 (10.1)	9 (10.0)
High alcohol intake (%)	55 (11.2)	79 (10.2)	68 (9.2)	39 (9.8)	15 (13.3)	23 (14.9)	2 (3.3)	4 (7.3)
Low-level physical activity (%)	448 (69.1)	744 (71.5)	738 (71.3)	428 (73.0)	132 (68.0)	149 (70.6)	70 (70.7)	64 (71.1)
DASH score (mean)	24.6 ± 5.6	24.5 ± 5.5	24.6 ± 5.5	24.1 ± 5.4	23.7 ± 5.3	23.4 ± 5.5	23.5 ± 5.4	23.3 ± 5.4
Poor diet quality^a^ (%)	255 (39.6)	422 (41.0)	412 (40.2)	260 (44.9)	93 (48.2)	109 (52.4)	49 (52.1)	46 (54.8)
BMI, kg/m^2^ (mean)	27.7 ± 4.6	28.1 ± 6.3	27.7 ± 4.6	29.0 ± 7.4	29.5 ± 4.8	31.0 ± 10.2	31.0 ± 4.6	31.3 ± 4.8
Overweight or obese^b^ (%)	479 (73.7)	786 (75.4)	769 (74.2)	470 (80.2)	163 (84.0)	190 (90.0)	88 (88.9)	83 (92.2)
Average systolic BP, mmHg (mean)	128.7 ± 18.1	128.4 ± 18.2	127.2 ± 17.9	128.3 ± 18.3	129.1 ± 17.8	134.2 ± 18.6	132.5 ± 19.1	134.5 ± 18.1
Average diastolic BP, mmHg (mean)	76.7 ± 10.0	76.4 ± 10.0	75.9 ± 9.8	76.2 ± 10.0	76.6 ± 10.1	79.2 ± 11.0	76.3 ± 9.9	77.0 ± 9.1
Hypertension^c^ (%)	280 (43.0)	472 (45.3)	455 (43.8)	301 (51.4)	109 (56.2)	135 (64.0)	72 (72.7)	66 (73.3)
Triglycerides, mmol/l (median)	1.1 (0.8─1.4)	1.1 (0.8─1.5)	1.1 (0.8–1.4)	1.1 (0.9–1.5)	1.2 (0.9─1.6)	1.3 (1.0–1.8)	1.6 (1.1–2.2)	1.7 (1.1–2.3)
High triglycerides^d^ (%)	110 (17.3)	168 (16.5)	155 (15.3)	98 (17.1)	40 (21.1)	56 (27.5)	44 (45.8)	43 (49.4)
HDL-C, mmol/l (mean)	1.51 ± 0.4	1.50 ± 0.4	1.51 ± 0.4	1.46 ± 0.4	1.41 ± 0.3	1.34 ± 0.4	1.20 ± 0.4	1.19 ± 0.4
Low HDL-C^e^ (%)	89 (14.0)	157 (15.4)	148 (14.6)	103 (18.0)	35 (18.4)	55 (27.0)	49 (51.0)	41 (47.1)
HbA_1c_, mmol/mol (median)	36.0 (35.0─37.0)	38.0 (36.0─39.0)	38.0 (36.0─40.0)	40.0 (39.0─42.0)	43.0 (42.0─44.0)	41.0 (38.0─43.0)	57.0 (50.0─69.0)	57.0 (49.0─69.0)
FPG, mmol/l (median)	4.9 (4.6─5.2)	5.0 (4.7─5.3)	4.9 (4.7─5.2)	5.2 (4.8─5.5)	5.4 (5.1─5.9)	5.9 (5.7─6.2)	8.0 (6.8─9.1)	8.4 (7.6─9.6)

Number and (%) are shown for dichotomous variables; DASH score, BMI, systolic BP, diastolic BP and HDL-C are shown as a mean (± one standard deviation). Age, triglycerides, HbA_1c_ and FPG are shown as a median (interquartile range). Numbers and % may vary as some variables have missing values.

^a^Bottom 40% DASH score.

^b^BMI ≥25.

^c^Systolic BP ≥140 or diastolic BP ≥90 or on anti-hypertensive medications.

^d^Triglycerides ≥1.7.

^e^HDL-C <1.03 males or <1.29 females.

Study participants diagnosed as pre-diabetic by FPG displayed a poorer metabolic profile compared with those classified using HbA_1c._ This poorer metabolic profile was characterised by higher BMI, systolic/diastolic BP and triglyceride levels, and lower HDL-C concentrations. Subjects diagnosed with type 2 diabetes displayed a metabolic profile that was broadly similar according to diagnosis by either assay.

### Univariate analyses

In univariate multinomial logistic regression analyses (Table 2), factors related to pre-diabetes, and directions and strengths of association, varied with the diagnostic method employed. Males displayed an almost two-fold (OR = 1.98, 95% CI: 1.46–2.69) increased odds of having pre-diabetes compared to females according to the FPG ADA criterion, whereas females were more likely to have pre-diabetes defined using HbA_1c_ ADA thresholds. Being married or cohabiting was protective against having pre-diabetes according to HbA_1c_ WHO-IEC and FPG ADA classifications and participants defined by both HbA_1c_ and FPG ADA criteria were also less likely to perceive their health as being very good or good. Subjects with a primary education only displayed a 61% (OR = 1.61, 95% CI: 1.15–2.26) increased odds of having pre-diabetes defined by FPG ADA classification. With regard to lifestyle behaviours, being a current smoker was significantly associated with pre-diabetes using HbA_1c_ ADA cut-offs while high alcohol intake and poor diet quality were associated with pre-diabetes defined using FPG. Associations with overweight/obesity, hypertension, high triglyceride and low HDL-C levels were noticeably significant and stronger among subjects diagnosed as having pre-diabetes by FPG. In general, participants identified by the FPG ADA pre-diabetes criterion displayed a profile more similar to patients diagnosed with type 2 diabetes than subjects identified as pre-diabetic according to HbA_1c_ ADA and WHO-IEC thresholds.

**Table 2:** Factors associated with pre-diabetes and type 2 diabetes according to diagnosis by HbA_1c_ or FPG.

Variable	Odds ratio (95% CI)											
	Pre-diabetes compared to normoglycaemia						Type 2 diabetes compared to normoglycaemia					
	HbA_1c_ ADA	P	HbA_1c_ WHO-IEC	P	FPG ADA	P	HbA_1c_ ADA	P	HbA_1c_ WHO-IEC	P	FPG ADA	P
Male	0.80 (0.64–0.99)	.045	1.03 (0.76─1.40)	.861	1.98 (1.46─2.69)	< .001	1.96 (1.25–3.07)	.003	2.20 (1.42─3.41)	< .001	2.45 (1.55─3.87)	< .001
Age ≥65 years	1.38 (1.10–1.74)	.006	1.62 (1.17─2.23)	.004	1.52 (1.12─2.07)	.008	2.60 (1.60–4.23)	< .001	2.41 (1.50─3.87)	< .001	2.63 (1.59─4.37)	< .001
Married or cohabiting	0.81 (0.62–1.07)	.138	0.63 (0.45─0.89)	.009	0.65 (0.47─0.91)	.013	0.93 (0.56–1.57)	.796	0.95 (0.58─1.58)	.853	0.84 (0.50–1.40)	.496
Health rating, very good or good	0.60 (0.43–0.84)	.003	0.75 (0.49─1.15)	.188	0.54 (0.37─0.79)	.001	0.27 (0.16–0.44)	< .001	0.33 (0.21─0.52)	< .001	0.32 (0.20─0.52)	< .001
Primary education only	1.26 (0.96–1.65)	.1	1.24 (0.87─1.79)	.239	1.61 (1.15–2.26)	.005	2.04 (1.27–3.28)	.003	1.89 (1.20─2.99)	.006	2.29 (1.42–3.68)	.001
Current smoker	1.67 (1.11─2.50)	.014	0.96 (0.55─1.67)	.887	1.05 (0.63–1.77)	.849	1.58 (0.77–3.26)	.215	1.20 (0.60─2.39)	.6	1.18 (0.57–2.43)	.652
High alcohol intake	0.86 (0.56–1.33)	.5	1.35 (0.75─2.43)	.324	1.73 (1.04–2.88)	.034	0.27 (0.07–1.15)	.076	0.30 (0.07─1.27)	.1	0.77 (0.27–2.21)	.632
Low-level physical activity	1.21 (0.95–1.55)	.132	0.85 (0.61─1.18)	.325	0.97 (0.70–1.34)	.841	1.08 (0.68–1.71)	.752	0.96 (0.61─1.51)	.861	0.99 (0.62–1.59)	.969
Poor diet quality^a^	1.24 (0.99–1.56)	.061	1.34 (0.98─1.82)	.063	1.64 (1.22–2.21)	.001	1.66 (1.08–2.57)	.022	1.57 (1.03─2.40)	.037	1.80 (1.15–2.82)	.01
Overweight or obese^b^	1.45 (1.11–1.89)	.007	1.71 (1.14─2.58)	.01	3.15 (1.97–5.05)	< .001	2.86 (1.49–5.47)	.002	2.61 (1.37─4.95)	.003	4.13 (1.89–9.05)	< .001
Hypertension^c^	1.40 (1.12–1.75)	.003	1.55 (1.14─2.11)	.005	2.28 (1.68–3.09)	< .001	3.53 (2.21–5.65)	< .001	3.23 (2.04─5.10)	< .001	3.52 (2.17–5.71)	< .001
High triglycerides^d^	0.99 (0.73–1.33)	.93	1.35 (0.92─1.98)	.13	2.10 (1.48–2.99)	< .001	4.04 (2.57–6.34)	< .001	4.28 (2.77─6.60)	< .001	5.43 (3.45–8.55)	< .001
Low HDL-C^e^	1.35 (0.99–1.84)	.059	1.24 (0.83─1.85)	.3	2.17 (1.52–3.09)	< .001	6.40 (4.04–10.12)	< .001	5.71 (3.70─8.82)	< .001	5.23 (3.31–8.24)	< .001

Univariate multinomial logistic regression.

^a^Bottom 40% DASH score.

^b^BMI ≥25.

^c^Systolic BP ≥140 or diastolic BP ≥90 or on anti-hypertensive medications.

^d^Triglycerides ≥1.7.

^e^HDL-C <1.03 males or <1.29 females.

### Multivariable analyses

In multivariable analyses (Tables 3–5), pre-diabetes was found to be significantly related to sex (HbA_1c_ ADA only), older age (HbA_1c_ ADA and WHO-IEC), health rating (HbA_1c_ ADA and FPG ADA), education (FPG ADA only), smoking (HbA_1c_ ADA only), poor diet quality (HbA_1c_ ADA only) and overweight/obesity (all definitions). Subjects classified by FPG ADA pre-diabetes thresholds displayed the least optimal metabolic profile defined by overweight and obesity (OR = 2.88, 95% CI: 1.53–5.43), hypertension (OR = 2.27, 95% CI: 1.51–3.40) and low HDL-C concentrations (OR = 1.75, 95% CI: 1.07–2.87).

**Table 3:** Multivariable analysis of factors associated with pre-diabetes according to diagnosis by HbA_1c_ using ADA classification.

Variable	Odds ratio	95% CI	P	Wald
Male	0.65	0.50─0.85	.001	10.37
Age ≥65 years	1.36	1.06─1.75	.017	5.70
Health rating, very good or good	0.53	0.36─0.77	.001	11.17
Current smoker	1.64	1.04─2.61	.034	4.47
Poor diet quality^a^	1.31	1.02─1.70	.038	4.30
Overweight or obese^b^	1.62	1.19–2.20	.002	9.51

^a^Bottom 40% DASH score.

^b^BMI ≥25.

**Table 4:** Multivariable analysis of factors associated with pre-diabetes according to diagnosis by HbA_1c_ using WHO-IEC classification.

Variable	Odds ratio	95% CI	P	Wald
Age ≥65 years	1.60	1.15─2.23	.005	7.83
Overweight or obese^a^	1.67	1.08─2.57	.02	5.40

^a^BMI ≥25.

**Table 5:** Multivariable analysis of factors associated with pre-diabetes according to diagnosis by FPG using ADA classification.

Variable	Odds ratio	95% CI	P	Wald
Health rating, very good or good	0.47	0.29─0.78	.003	8.82
Primary education only	1.76	1.13–2.73	.012	6.32
Overweight or obese^a^	2.88	1.53─5.43	.001	10.67
Hypertension^b^	2.27	1.51─3.40	< .001	15.56
Low HDL-C^c^	1.75	1.07─2.87	.026	4.96

^a^BMI ≥25.

^b^Systolic BP ≥140 or diastolic BP ≥90 or on anti-hypertensive medications.

^c^HDL-C <1.03 males or <1.29 females.

## Discussion

In this study of middle- to older-aged Irish men and women we used HbA_1c_ ADA (5.7%–6.4%), HbA_1c_ WHO-IEC (6.0%–6.4%) and FPG ADA (5.6–6.9 mmol/l) criteria to diagnose pre-diabetes. Stronger associations with diabetes-related phenotypes such as overweight/obesity, hypertension, high triglyceride and low HDL-C concentrations were found when using FPG compared to HbA_1c_ thresholds for diagnosing pre-diabetes. Our findings also demonstrate considerable discordance in pre-diabetes prevalence estimates according to method of diagnosis. In addition, as different factors were selected in forward elimination regression models, our results also indicate that the profile of patients identified vary considerably according to the method of pre-diabetes classification employed.

Identifying individuals at risk of type 2 diabetes development is particularly relevant given the increasing prevalence of diabetes worldwide, frequent diabetes-related complications seen at diagnosis and the increased cardiovascular disease risk and mortality associated with diabetes [43]. Consequently, identifying factors associated with pre-diabetes is important for recognising patients who should be targeted and as to which lifestyle modifications or medical interventions should be employed. Though few prospective studies have comprehensively identified features related to pre-diabetes development, it has been suggested that factors associated with pre-diabetes mirror those for type 2 diabetes [1, 2, 4, 44], as is indicated in our study. Results from our research demonstrate that subjects diagnosed with pre-diabetes using FPG ADA cut-offs were more likely to be male and to have a primary level education only, findings which were also observed among participants diagnosed with type 2 diabetes by all criteria. Previous studies in Ireland have found the prevalence of diabetes to be consistently higher in males compared to females [8]. Evidence suggests that men are at a higher risk of developing type 2 diabetes as they develop diabetes at a lower BMI, are more predisposed to central fat deposition and are more prone to insulin resistance [45]. Previous research has also indicated that diabetes cases occur disproportionately amongst individuals who are economically deprived and have lower educational levels [3, 46].

Studies have additionally shown that changes in lifestyle behaviours in people at risk of developing diabetes, in particular modifications in dietary intake, may prevent or delay its onset [7, 13, 43]. A meta-analysis of prospective cohort studies which examined dietary effects in preventing diabetes concluded that several diets, including the DASH diet, were associated with a 20% decrease in the risk of future type 2 diabetes [47]. Although uncertainty exists regarding the mechanisms that explain the association between dietary intake and type 2 diabetes, the DASH diet emphasis on fruits and vegetables, lower-fat dairy foods and reduced consumption of red meat and sugars may be beneficial for patients who are aiming to lose weight or maintain their weight at a healthy level [48]. Furthermore, adherence to the DASH diet is associated with a reduction in systolic blood pressure [39] and has also been observed to improve conventional lipid profiles [49, 50] and lipoprotein subclass concentrations [51]. Overweight and obesity, hypertension, raised triglyceride and low HDL-C levels are well established risk factors for type 2 diabetes [3] and these variables showed noticeably significant and stronger associations with pre-diabetes defined by FPG ADA thresholds in our sample. Importantly, it has been observed that subjects with a combination of these features have a five-fold increased risk of developing diabetes [52].

The prevalence of pre-diabetes was found to 43.9% (95% CI: 41.2%─46.5%), 14.5% (95% CI: 12.7%─16.5%) and 15.8% (95% CI: 13.9%─17.8%) in our sample according to HbA_1c_ ADA, HbA_1c_ WHO-IEC and FPG ADA definitions, respectively. International inconsistencies in the use of different definitions in terms of both glucose measures and cut-points are thus likely to have a major effect on pre-diabetes prevalence estimates and should therefore be considered when comparing findings from different regions [12]. In Europe, using HbA_1c_ WHO-IEC cut-offs, Bonaldi et al. reported a pre-diabetes prevalence of 3.0% (95% CI: 1.7%–5.0%) in France among adults aged 55–74 years in 2007 [53]. Rathmann et al. reported a pre-diabetes prevalence of 23.0% in a population aged 55–74 years in Germany during 2000 using WHO definitions of impaired fasting glucose or impaired glucose tolerance [54]. Using HbA_1c_ ADA cut-offs, a study conducted in the United Kingdom estimated the prevalence of pre-diabetes in 2011 to be 48.7% (95% CI: 46.6%‒50.8%) among subjects aged 40+ years [55]. In a 2016 multi-ethnic study of pre-diabetes in low-to-middle income countries (mean age: 47.7 ± 14.0 years; 45.9% male), the prevalence of pre-diabetes was found to be 17.8% (95% CI: 17.0%–18.7%) in the Southern Cone of Latin America, 17.1% (95% CI: 15.9%–18.5%) in Peru, 24.0% (95% CI: 23.2%–24.7%) in South Asia and 9.9% (95% CI: 8.3%–11.8%) in South Africa using the FPG ADA pre-diabetes criterion [56].

As previously discussed, discordance in pre-diabetes prevalence estimates according to diagnostic criteria is well-documented [7, 23, 57] and was also noted in the first wave analysis of the Mitchelstown Cohort in 2010/11, where pre-diabetes prevalence was found to be 49.1% and 11.5% using HbA_1c_ ADA and FPG ADA thresholds, respectively [6]. A higher prevalence using HbA_1c_ was also found in a Canadian study published in 2015. In this research, the prevalence of pre-diabetes using HbA_1c_ ADA cut-offs was 33.1% compared 13.3% using FPG ADA cut-points [9]. Results consistent with our findings were also found in a Spanish study (21.7% using HbA_1c_ vs. 16.3% using FPG) [57] and one conducted in Palestine (45.8% using HbA_1c_ vs. 24.6% using FPG) [23]. In contrast, some studies have found a higher prevalence among those diagnosed with FPG compared to those identified using HbA_1c_. Research conducted in the United States between 2005─2008 found the prevalence of pre-diabetes to be 14.2% by HbA_1c_ compared to 26.2% by FPG [14]. Another American study found similar results: 12.6% using HbA_1c_ vs. 28.2% by FPG [21]. Evidence suggests that disparities between HbA_1c_ and FPG may be attributable to sex, age or ethnic differences in the populations studied [6, 13, 14, 21, 22].

Although a more expensive test, perceived benefits of the use of HbA_1c_ measurement, over FPG, include greater pre-analytical stability, lower biological variability and that the assay may be performed in non-fasting blood samples [6, 23]. Nevertheless, although prevalence figures for type 2 diabetes among middle-aged Irish adults using HbA_1c_ were previously found to be comparable between our sample, the SLÁN survey and the TILDA study, prevalence estimates for pre-diabetes vary widely [1, 6, 12]. These findings are important regarding future diabetes estimates within Ireland, as accurate assessment of progression rates to type 2 diabetes is needed for efficient allocation of resources in order to optimise public health prevention strategies [6]. Crucially, differences in metabolic profiles among patients identified, and extensively varying pre-diabetes prevalence estimates observed in Ireland and other countries, suggest that HbA_1c_ may lack both validity and reliability as a tool for identifying patients at risk of type 2 diabetes development. Standardisation issues with regard to different procedures used for assessing HbA_1c_ levels may still need to be addressed [6]. Our study will inform national decision-makers on which definition of pre-diabetes to use for monitoring trends in prevalence over time.

### Strengths and limitations

This study has several strengths. Firstly, it provides estimates of the prevalence of pre-diabetes in an older Irish population using recently acquired data. Additionally, the study reported pre-diabetes prevalence using three different criteria to emphasise the discordance between diagnostic methods. Our results are of potential clinical significance in terms of early detection of pre-diabetes and the use of pre-diabetes classification as a method for determining diabetes risk. An optimal procedure for prediction of type 2 diabetes development within clinical practice is important for timely intervention in order to avoid the complications associated with the disease.

Despite these strengths, several limitations should be noted. The cross-sectional study design limits inference with regard to causality and precludes drawing conclusions regarding the temporal direction of relationships. Moreover, the use of self-reported questionnaires is subject to potential inaccuracies, recall and reporting bias and residual confounding arising from imprecise measurement of variables should also be considered. Furthermore, our data were derived from a single primary care-based sample. Although results from the Mitchelstown Cohort Study demonstrate prevalence rates for obesity and cardiovascular outcomes similar to those observed in other nationally representative Irish studies [3, 6, 58], the possibility that this sample is not representative of the source population must be acknowledged. However, previous research suggests that approximately 98% of Irish adults are registered with a GP and that, even in the absence of a universal patient registration system, it is possible to perform population-based epidemiological studies that are representative using our methods [59]. In addition, Ireland presents a generally ethnically homogeneous population [60]. Thus, the relationships we have observed may be comparable to other Irish adults. As random sampling of subjects and the use of validated methods for data collection ensured internal sample validity, it is equally possible that the relationships described may be generalisable to a similar middle- to older-aged white European population. Nevertheless, further research to confirm these findings is warranted.

## Conclusions

In summary, our results agree with findings from previous research indicating considerable discordance in pre-diabetes prevalence estimates according to diagnostic method. The profile of patients identified as having pre-diabetes also varied considerably by diagnostic criteria employed. This discordance is concerning and will have important implications for prevention planning. A National Diabetes Prevention Programme is currently being developed in Ireland. Monitoring the prevalence of pre-diabetes over time will be important to assess the effectiveness of this programme. As an agreement will need to be reached on which definition of pre-diabetes to use for monitoring purposes, our study may help inform this decision. Further research is required to determine which diagnostic method is superior and should be recommended to healthcare providers in a primary care setting.

## Supporting information

S1 File Dataset. The Mitchelstown Cohort Rescreen Study dataset.
(SAV)
